# Metformin inhibited esophageal squamous cell carcinoma proliferation *in vitro* and *in vivo* and enhanced the anti-cancer effect of cisplatin

**DOI:** 10.1371/journal.pone.0174276

**Published:** 2017-04-13

**Authors:** Feng Wang, Xianfei Ding, Tao Wang, Zhengzheng Shan, Jun Wang, Shaoxuan Wu, Yanyan Chi, Yana Zhang, Zhuan Lv, Liuxing Wang, Qingxia Fan

**Affiliations:** Department of Oncology, First Affiliated Hospital of Zhengzhou University, Zhengzhou, Henan Province, China; University of South Alabama Mitchell Cancer Institute, UNITED STATES

## Abstract

Esophageal squamous cell carcinoma (ESCC) is an aggressive malignancy with poor prognosis in China. Chemotherapy now is one of the most frequently used treatments for patients with ESCC in middle or late stage, however the effects were often limited by increased chemoresistance or treatment toxicity. So it is urgent to find new drugs to treat ESCC patients. Metformin with low cost and toxicity has proved to have anti-cancer effects in a numerous cancers, while its role and mechanism in ESCC has seldom been studied. In the present study, we found that metformin exhibited not only an anti-proliferation ability in a dose and time dependent manner but also a proapoptosis effect in a dose dependent manner in ESCC cell line KYSE450. Our in vivo experiment also showed that metformin markedly inhibited KYSE450 xenograft tumors growth compared to those treated with normal saline. What’s more, no obvious toxic reactions were observed. To further explore the underlying mechanism, we found that metformin treatment could significantly damp the expression of 4EBP1 and S6K1 in KYSE 450 cells in vitro and in vivo, furthermore, the p-4EBP1 and p-S6K1 expression in KYSE 450 cells were also inhibited greatly in vitro and in vivo. During the therapy of cancer, in order to overcome side effects, combination therapy was often used. In this paper, we demonstrated that metformin potentiated the effects of cisplatin via inhibiting cell proliferation and promoting cell apoptosis. Taken together, metformin owned the potential anti-cancer effect on ESCC in monotherapy or was combined with cisplatin and these results laid solid basis for the use of metformin in ESCC.

## Introduction

Esophageal cancer (EC) is a worldwide problem of public health [1]. The American Cancer Society estimates that there will be 16910 new cases and 15690 deaths in the United States in 2016 [2, 3]. Because of EC’s metastasis at its early stage and mild symptoms, once diagnosed, most patients were in their middle and late stage and lost the best time for surgery. So although the incident for EC ranks the 6^th^ among the digestive system cancer, its mortality rate ranks the 4^th^ and the 5 year survival rate is less than 20% [4, 5]. Chemotherapy becomes the main treatment for these patients in their middle and late stage, while the adverse effects for chemotherapy is very big and some patients can’t bear it. Most of all, some patients are easy to produce drug resistance during chemotherapy [6, 7]. Therefore, it is urgent to find new drugs or methods for patients with EC.

Metformin, a widely used drug for treatment of type 2 diabetes, now has proved to have chemopreventive effects on cancers. Many studies have shown that cancer in diabetics treated with metformin have a lower incidence and mortality rate than those without [8, 9]. For example, a study done by a Dutch labor prospective observational trial found that use of metformin for cancer patients was associated with lower mortality in a dose-dependent manner after they followed 1300 patients for about 9 years [10]. In addition, Bowler et al also found lower mortality rate for patients with metformin verse those with sulfonylurea [11]. A recent retrospective study with 196 patients reported that the overall survival rate of EC patient with long-term treatment of metformin was all higher, while the metastasis rate was lower than those without treatment of metformin [12–13]. Furthermore, a number of studies have confirmed that metformin inhibited the proliferation ability of EC, lung cancer, gastric cancer and others in vitro and in vivo [14, 15]. However, the molecular mechanisms of the anti-cancer effects of metformin have not been fully elucidated.

Some researchers demonstrated that metformin did their works by inhibiting NF-κB and STAT3 activities in cancer cells. While some others showed that metformin perhaps played an important role in cancer progression via regulating the mTOR signaling pathway. mTOR signaling pathway plays a critical role in cancer progression, resistance to chemotherapy and poor prognosis by modulating the activation of many target genes [16]. 4E-binding protein 1 (4EBP1) and the p70 ribosomal S6 kinases 1 (S6K1) are two important downstream effectors of the mTOR signaling pathway and involved in regulation of the translational machinery [17]. They are always co-expressed and up-regulated in cancer cells [17]. In some kinds of cancers, metformin could dampen tumorigenicity via inhibition of mTOR signaling pathway [18–20]. However, whether metformin could depress the progression of EC by inhibiting the phosphorylation of 4EBP1 and S6K1 has not been investigated until now.

EC is categorized into esophageal adenocarcinoma and esophageal squamous cell carcinoma (ESCC), while ESCC is the most common type in China. In this study, we demonstrated that metformin inhibited the proliferation ability and increased the apoptosis ability of ESCC cell line: KYSE450 in vitro and in vivo. Metformin also potentiated the effects of cisplatin, a commonly used drug in chemotherapy. Additionally, metformin could exert its effects via decreasing the phosphorylation of 4EBP1 and S6K1. So metformin might be a promising drug for patients with ESCC.

## Materials and methods

### Reagents and antibodies

Metformin was purchased from Sigma Co. Antibodies against 4EBP1 and S6K1 were from Abcam Co. MTT and Annexin V-FITC/PI apoptosis detecting kit were from Nanjing Kaiji Biotech Co. Trizol regants, TransScript First-strand cDNA Synthesis SuperMix kit and PCR amplification kit were all from Beijing Solarbio Co. Other chemicals were purchased from Shanghai Sango Co.

### Cell line and cell culture

Human ESCC cell line: KYSE450 which was authenticated by STR analysis was kindly given by the Cancer Hospital of Chinese Academy of Sciences. KYSE 450 cells were grown in RPMI 1640 medium supplemented with 100IU/mL penicillin, 100μg/mL streptomycin and 10% fetal bovine serum (FBS) at 37°C in a humidified atmosphere containing 5% CO_2_. All experimental cells were in logarithmic growth phase.

### MTT assay

For the determination of cell proliferation, KYSE450 cells were digested by 0.25% trypsin and plated in 96 well plates at a density of 5×10^3^. The cells adhered to the bottom of each well after 24h since cultivation, and then 5, 10, 20 and 40mmol/L metformin were added. Each group has 5 multiple holes. After cultivation for 24, 48, and 72h, 20μL 3-(4,5-dimethyl-2-thiazol)-2,5-diphenyl-2H-tetrazolium bromide(MTT) (5g/L) were added in each group and incubated for another 4h. Then 150μL DMSO was added, and the absorbance was measured at a wavelength of 490 nm. The absorption data (AD) were used to calculate the inhibitory rate on proliferation of metformin. Inhibitory rate = (AD _blank control_–AD _experiment group_)/ AD _experiment group_×100%. Experiments were repeated at least 3 times. To detect the combination effects of metformin and cisplatin on the proliferation of KYSE450, 5, 10, 20 and 40mmol/L metformin combined with 2mg/L cisplatin were added and cultivated for24 and 48h. 4h after added of MTT, DMSO was used and absorbance was measured at a wavelength of 490 nm. Inhibitory rate was calculated using the above formula.

### Annexin V-FITC/PI apoptosis assay

For the determination of cell apoptosis, KYSE450 cells were digested by 0.25% trypsin and plated in 6 well plates at a density of 1.5×10^5^. After cultivating for 24h without FBS, RPMI 1640 with 0, 5, 10 and 20 mmol/L metformin or RPMI 1640 with 0, 5, 10 and 20 mmol/L metformin combined with 2mg/L cisplatin were used to cultivate KYSE450 cells for another 48h. Then KYSE450 cells were harvested, washed with PBS and resuspended in 0.5 mL PBS. Followed by addition of 300 μL Annexin V, 5 μL Annexin V-FITC and 5 μL PI, the apoptosis rate of KYSE450 were estimated by using Annexin V-FITC/PI apoptosis detection kit according to the manufacturer's protocol. KYSE450 treated without annexin V-FITC, PI were used as negative control. The apoptosis rate of KYSE450 (%) = apoptotic cells/ total cells×100%.

### Real time RT-PCR assay

KYSE450 cells treated with 5, 10 and 20 mmol/L metformin for 48h and KYSE450 cells treated with 20 mmol/L metformin for 24, 48 and 72 h were harvested respectively. KYSE450 cells without treatment were used as blank control. Total RNA was extracted by Trizol according to the manufacturer's protocol and ultraviolet spectrophotometric method was used to detect the purity and concentration of the exreacted RNA. Then cDNA was generated from 1 μg total RNA by using transScript First-strand cDNA Synthesis SuperMix kit following the instruction manual. Real time PCR analysis was carried out with the SYBR^®^ Green PCR Master Mix with the following primers: 4EBP1 upper primer: 5´- GGAGTGTCGGAACTCACCTG -3´, lower primer: 5´- ACACGATGGCTGGTGCTTTA -3´, with amplified fragment length of 195bp; S6K1 upper primer: 5´-GGACATGGCAGGAGTGTTTG-3´, lower primer: 5´- TTTCTGGCCCTCTGTTCACA-3´, with amplified fragment length of 188. The reaction conditions for PCR were: 94°C for 5 min, 30 cycles with 94°C for 30 s, 54°C (4EBP1/S6K1) for 30 s and 72°C for 30 s, at last extension at 72°C for 5 min. The values of 4EBP1 and S6K1 were normalized to the relative amounts of GAPDH. All experiments were repeated three times.

### Western blot assay

Briefly, KYSE450 cells treated with 5, 10 and 20 mmol/L metformin for 48h and KYSE450 cells treated with 20 mmol/L metformin for 24, 48 and 72 h were harvested as described previously and lysed by lysis buffer. The protein concentration in each group were determined by BCA kits. After heated at 100°C for 10 min, 80μg protein lysates in each group were separated on SDS/PAGE gels and transferred for to PVDF membranes by using semi-dry transfer methods. Then the membranes were blocked by 5% (w/v) non-fat milk in PBS containing 0.1% Tween-20 for 2h and incubated with primary antibody overnight. After washed by TBST, the membranes were incubated with secondary antibody. Immunoreactive products were revealed by chemiluminescence, normalized with β-actin and then quantified by using Image J. All experiments were repeated three times.

### Animal study

All animal in the experiment were approved by the Ethical Committee of the First Affiliated Hospital of Zhengzhou University. 15 Male BALB/c nude mice 4–6 weeks old were purchased from Shanghai Institute of Biology and allowed to be accustomed to pathogen free new environment for about one week before experiment. Then 0.2 ml KYSE450 cells suspended in PBS at a density of 7.5×10^7^ were injected into each SCID mice subcutaneously in the flank back skin. After about 5 days, xenograft tumors about bean size could be touched at the inoculation site. 10 days later, 10 mice with about the same volume of xenograft tumors were selected and randomly assigned to two groups (n = 5). One group were injected with 0.15mL.metformin (35.75 mg/kg/d) by intraperitoneal injection, the other one were injected with the same volume normal saline for 15 consecutive days. Tumor volumes were routinely measured every 3 days by width and length using caliper. Tumor volume = (length×width^2^)/2. At the end of treatment, 10 mice had been in carbon dioxide anesthesia for 10 min, and after no activity was found the nude mice would be taken out to confirm that they were dead. For those which could not be sure that they were dead, cervical dislocation were performed for them. Then the xenograft tumors were taken out and weighed quickly. Then they were fixed in 10% (v/v) neutral buffered formalin and embedded in paraffin for immunohistochemistry analyses.

### Immunohistochemistry assay

Paraffin xenograft tumor tissue sections were cut at 4 μm and paraffin was removed using different concentration of xylene series and rehydrated with graded ethanol series. Then epitope retrieval was done by boiling the tissue sections on slides in sodium citrate buffer in pressure cooker. After incubated with 3% H_2_O_2_ to eliminate the endogenous peroxidase activity, the slides were blocked with 5% (v/v) normal goat serum for 1h and incubated with primary antibodies at 4°C overnight. After washed with TBST for 3–4 times, HRP-linked secondary antibody was added for 40 minutes. Signals were detected by 3,3'-diaminobenzidine (DAB) substrate kit and counterstained by hematoxylin. Images were captured at × 200 magnification. Expression of 4EBP1 and S6K1 could be found in the cytoplasm or nucleus, while the p-4EBP1 and p-S6K1 were mainly found in the nucleus. Image-Pro Plus was used to analyze the average density of each image of immunohistochemistry.

### Tunnel assay

Paraffin xenograft tumor tissue sections were cut at 4 μm and paraffin was removed using different concentration of xylene series and rehydrated with graded ethanol series. Then 20ug/ml protease K was added to remove the tissue protein. The slides were washed by PBS for two times, then 2 drops of TdT were added for 1-5min at room temperature. 54ìl TdT added again on the slides and cultured for 1h at 37°C. Slides without TdT were used as control. After washing by PBS, peroxidase labeled anti digoxin antibody were added and cultured again for 30 min at 37°C. DAB was added at last to show color. And then the apoptotic rate was measured. The percentage of the average number of apoptotic cells in 7 positive visual field (200 cells were calculated in each field) were viewed as apoptotic rate.

### Statistical analysis

The Student t-test was applied to evaluate the differences between the experiments and control groups. P-values <0.05 was considered to be statistically significant.

## Results

### Metformin inhibited the proliferation ability of KYSE450 cells and promoted KYSE450 cells apoptosis

**Fig 1:**
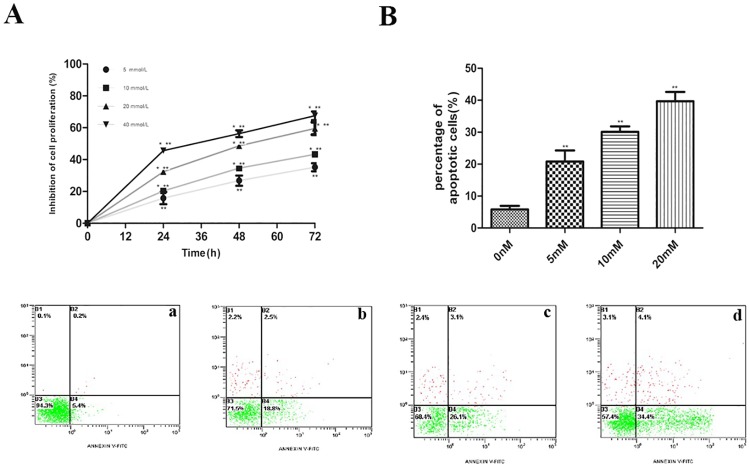
Effects of metformin on the proliferation and apoptosis ability of KYSE 450 cells. A. KYSE 450 cells treated with 5, 10, 20 and 40mmol/L metformin for 24, 48, 72h and cell proliferation was measured by MTT assay. The results revealed that the inhibition rate of the proliferation ability of KYSE 450 cells increased significantly with the time going and concentration rising. *P<0.01 denotes a significant difference between different concentration at the same time point; **P<0.05 denoted a significant difference between different time point at the same concentration. B. Annexin V-FITC/PI was used to analyze of apoptosis ability of KYSE 450 cells treated with 0, 5, 10 and 20 mmol/L metformin. With the increase of concentration, the percentage of apoptotic cells improved markedly. a was the control group, b-d were the group of 5, 10, 20 mmol/L metformin respectively. **P<0.05 denoted a significant difference between different concentration.

As we have described above, metformin can affect the proliferation ability of different cancer cells. In order to investigate the effect of metformin on KYSE450 cells, KYSE450 cells were cultured in RPMI 1640, in the presence (5, 10, 20, 40 mmol/L) for 24, 48 and 72h respectively. With the increase of drug concentration and the prolongation of action time, the inhibition effect of metformin on the proliferation of KYSE450 cells was gradually increased (Fig 1A). These data indicated that in ESCC cells, metformin also had anti-proliferation effects. This anti-proliferation effect was in a dose and time dependent manner.

We have determined a dose and time dependent manner of metformin on the proliferation ability of KYSE450 cells. To further investigate the anti-cancer effect of metformin on KYSE450 cells, we determine the apoptotic effect of metformin on KYSE450 cells. The number of apoptotic cells was quantified, and the results were shown in Fig 1B. Treatment of KYSE450 cells with metformin (0, 5, 10, 20 mmol/L) caused a significantly increase in the percentage of apoptotic cells and it was in a dose dependent manner. When KYSE540 cells were treated with 5, 10, 20 mmol/L metformin, the percentage of apoptotic cells were (20.8±1.4)%, (30.1±1.6)%, (39.7±1.4)% respectively, and there had significant increase compared to control cells (5.8±1.9)%. So, these results demonstrated that metformin induced apoptosis of KYSE450 cells.

### Metformin exerted its anti-cancer effect via activation of 4EBP1 and S6K1 in a dose and time dependent manner

**Fig 2:**
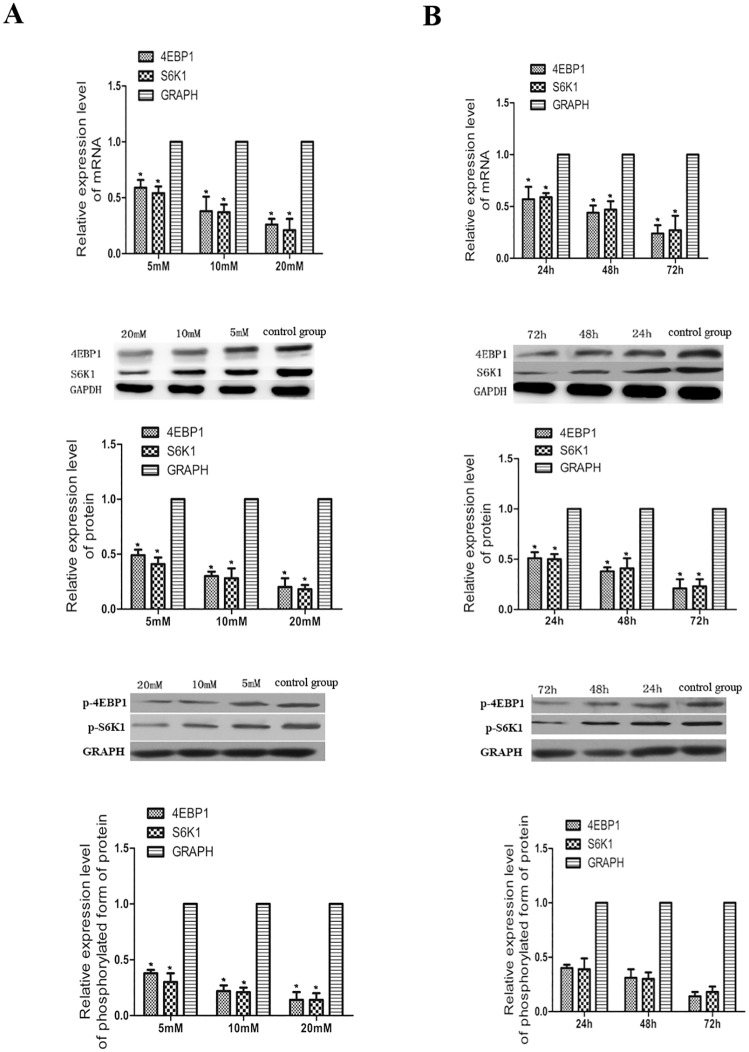
Metformin treatment inhibited the expression of 4EBP1 and S6K1. **A**. KYSE 450 cells were treated with 5, 10, 20 mmol/L of metformin for 48h. RT-PCR and western blot results showed that with the increase of concentration, the mRNA and protein expression of 4EBP1and S6K1 decreased significantly compared to those untreated KYSE 450 cells. Furthermore the protein expression of phosphorylated form of 4EBP1 and S6K1 were also inhibited by metformin in a dose dependent manner. **B.** KYSE 450 cells were treated with 20 mmol/L of metformin for 24, 48 and 72h respectively. RT-PCR and western blot results showed that with the extension of time, the mRNA and protein expression of 4EBP1and S6K1 also decrease greatly compared to those untreated KYSE 450 cells. In addition, the the protein expression of phosphorylated form of 4EBP1 and S6K1 were also inhibited by 20 mmol/L metformin in a time dependent manner. *P < 0.05 denoted a great different effects of different concentrations, different time of metformin on the 4EBP1 and S6K1 mRNA, protein or phosphorylated form of protein expression for esophageal cancer cells KYSE450.

4EBP1 and S6K1 were two target genes of mTOR signaling pathway. mTOR through cooperating with PI3K-dependent effectors to phosphorylate 4EBP1 and S6K1. Deregulation of multiple elements of the mTOR signaling pathway, especially, the overexpression of 4EBP1 and S6K1 had been reported in many kinds of cancer, and the overexpression of these major components of the mTOR signaling pathway had a remarkably effects on tumour progression. In order to investigate the mechanism for the anti-cancer effect of metformin on KYSE450 cells, we examined the effects of metformin treatment on the expression levels of 4EBP1 and S6K1 by real time RT-PCR and western blot assays. The results showed that treatment of KYSE450 cells with 5, 10, 20 mmol/L metformin resulted in a remarkable decrease of the expression levels of 4EBP1 and S6K1 in a dose dependent manner (Fig 2A). Furthermore, after the KYSE450 cells were treated with 20 mmol/L metformin for 24, 48, 72h, the expression levels of 4EBP1 and S6K1 were also dampened greatly in a time dependent manner (Fig 2B). As we had suggested before, 4EBP1 and S6K1 always did their after they were phosphorylated. So we then used WB again to test the expression levels of p-4EBP1 and p-S6K1 in KYSE450 cells treated with 5, 10, 20 mmol/L metformin or 20 mmol/L metformin for 24, 48, 72h. And the results showed that metformin treatment also inhibited the expression of p-4EBP1 and p-S6K1. These results indicated that metformin induced anti-cancer effect perhaps via inhibiting the expression of 4EBP1 and S6K1.

### Metformin inhibited tumor growth in vivo

**Fig 3:**
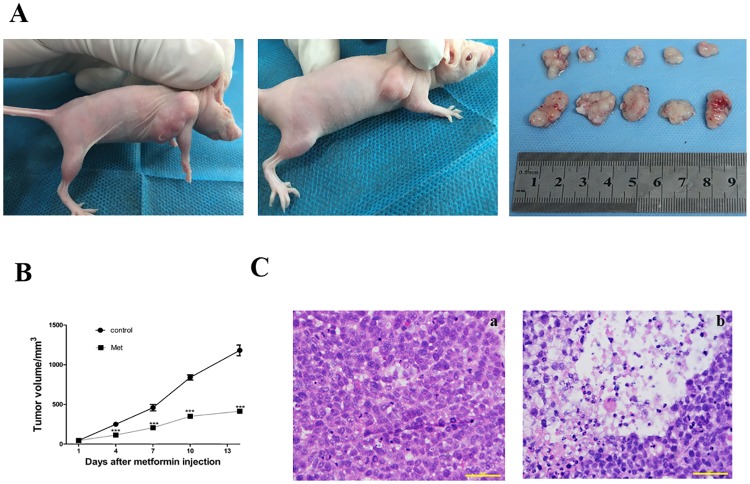
Metformin inhibited tumor growth in vivo. **A.**KYSE 450 xenograft treated with metformin (35.75 mg/kg/d) for 15 days were smaller than those treated with normal saline. **B.** Tumor volume were significantly reduced in metformin treated group than control group. ***P < 0.0001, denoted a markable difference between the size and volume of tumor-bearing nude mice of control group and metformin group. **C.** HE stain showed that the area of necrosis in xenograft tumors derived from metformin treated group were about 50% larger than those derived from control group.

After subcutaneously inoculated of KYSE450 cells for 7d, the volume of the xenograft tumors could reach 100 mm^3^, and the tumor formation rate was up to 80%. Then the male nude mice with xenograft tumors was randomly divided into two groups (n = 5). One group was treated with metformin, the other was treated with normal saline as control from the 8^th^ day after inoculation. This procedure lasted for 15 days. After xenograft tumors were taken out, it was obvious that those treated with metformin for consecutively 15 days were smaller than those treated with normal saline (Fig 3A). The tumor growth was inhibited about 70% in metformin treated group compared to those in control group (Fig 3B). In addition, treatment of metformin at the selected dose was well tolerated by mice. Other side effects were also not noted. Besides obvious reduction of growth, histological examination of excised xenograft tumors revealed that the area of necrosis in xenograft tumors derived from metformin treated group were about 50%, significantly higher than those in control group (Fig 3C). The results of TUNNL showed that the rate of apoptosis in metformin group was greater than those in control group. And the apoptotic rate in metformin treated groups were (43.5±1.65)% while the apoptotic rate in normal saline treated groups were only (22.3±2.12)% (p<0.05). Taken together, metformin inhibited xenograft tumor growth in vivo.

### Metformin inhibited the expression of 4EBP1 and S6K1 in vivo

**Fig 4:**
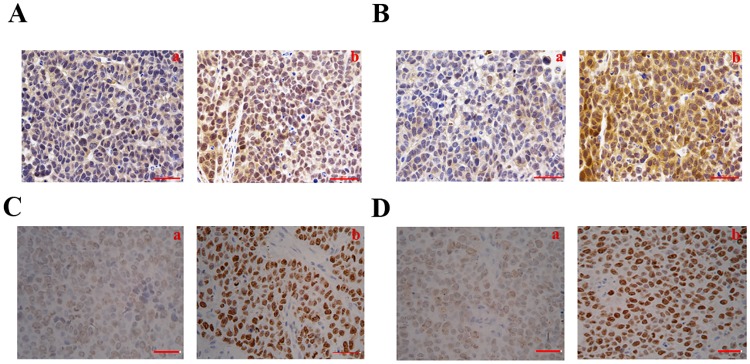
Metformin inhibited 4EBP1 and S6K1 expression in vivo. Xenograft tumors were harvested, fixed and paraffin-embedded, and stained for 4EBP1, S6K1, p-4EBP1 and p- S6K1 by immunohistochemistry. The protein expression of 4EBP1 and S6K1 were mainly located in the nuclear and cytoplasm of the KYSE450 cells. Immunohistochemical examination exhibited decreased average density of stain for 4EBP1 and S6K1 in metformin treated group than those in control group. The protein expression of p-4EBP1 and p-S6K1 were mainly located in the nuclear of the KYSE450 cells. And the immunohistochemical examination exhibited decreased average density of stain for p-4EBP1 and p-S6K1 in metformin treated group than those in control group. **A.** The protein expression of 4EBP1 in metformin group (a) and control group (b). **B.** The protein expression of S6K1 in metformin group (a) and control group (b). **C.** The protein expression of p-4EBP1 in metformin group (a) and control group (b). **D.** The protein expression of p-S6K1 in metformin group (a) and control group (b).

To further investigate the mechanism of metformin’ anti-cancer effect, immunohistochemistry analysis was done on excised xenograft tumors from mice treated with metformin or normal saline. Consistent with the results in vitro, the expression of 4EBP1 and S6K1 were all markedly reduced in tumors treated with metformin than those treated with normal saline (Fig 4A, 4B, 4C and 4D). This suggested that metformin could modulate the expression of 4EBP1 and S6K1. The anti-cancer effect of metformin perhaps were performed via decreasing the expression of 4EBP1 and S6K1.

### Metformin enhanced the anti-proliferation effect and promoted the proapoptotic role of cisplatin in KYSE450 cells

**Fig 5:**
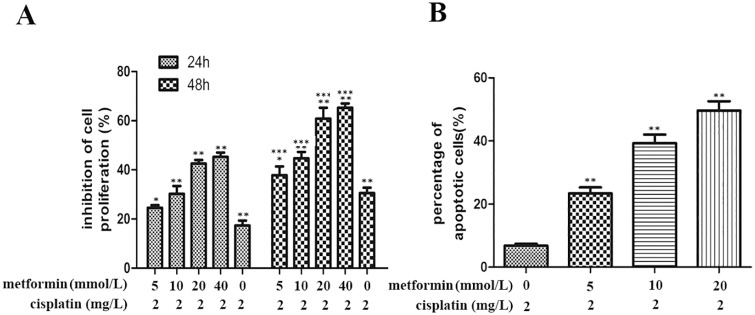
The combination effects of metformin and cisplatin on the proliferation and apoptosis ability of KYSE 450 cells. A. KYSE 450 cells treated with 5, 10, 20 and 40mmol/L metformin combined with 10ug/ml cisplatin for 24, 48h and cell proliferation was measured by MTT assay. The results showed that metformin enhanced the inhibition rate of cisplatin on the proliferation of KYSE450 cells. Furthermore, the inhibition rate increased with the increase of the concentration of metformin. *P<0.01 or **P<0.05 denotes a significant difference between different concentration at the same time point; ***P<0.05 denoted a significant difference between different time point at the same concentration. B. Annexin V-FITC/PI was used to analyze of apoptosis ability of KYSE 450 cells treated with 0, 5, 10 and 20 mmol/L metformin and cisplatin(10ug/mL) at the same time. The results demonstrated that metformin could also improve the apoptosis caused by cisplatin and the percentage of apoptotic cells increased with the rising concentration of metformin. **P<0.05 denoted a significant difference between different concentration.

Cisplatin, a well-known chemotherapeutic drug, has been used to treat a numerous cancers including ESCC with some side effects—drug resistance and increased toxicity. Therefore, the combination of cisplatin with other drugs have been considered to overcome these side effects. MTT assay was used to check anti-proliferation effects of combination of metformin (5, 10, 20, 40 mmol/L) and cisplatin (2mg/L) at 24, 48 and 72h respectively. The results showed that combining metformin and cisplatin together exerted greater anti-proliferation effects than did the use of metformin or cisplatin alone (Fig 5A).

With the increase of drug concentration and the prolongation of action time, the inhibition effect on the proliferation of KYSE450 cells of metformin combined cisplatin was gradually increased (Fig 5A). These data indicated that in ESCC cells, metformin could increase the anti-proliferation effect of cisplatin. This anti-proliferation effect was in a dose and time dependent manner. Combination of metformin and cisplatin could be used to treat patients with ESCC.

Cisplatin treatment now is the basis of treatment of many cancers. However, as we have said before, drug resistance has been identified in many patients with ESCC. And one of the reasons for cisplatin resistance was anti-apoptotic mechanisms. Metformin used alone increased the apoptotic rate of KYSE450 cells. In order to observe whether or not the apoptotic rate of KYSE450 cells could be promoted after being treated with metformin and cisplatin together, Annexin V-FITC/PI apoptosis assay was used. As shown in Fig 5B, the treatment of KYSE450 cells with metformin (0, 5, 10, 20 mmol/L) and cisplatin(2mg/L) together caused a markedly increase in the percentage of apoptotic cells and it was in a dose dependent manner. So combination of metformin and cisplatin might be an ideal treatment of ESCC.

## Discussion

With the increased incidence and mortality of ESCC in China, and the toxic and negative effect of chemotherapy for patients in middle and late stages, it is urgent to find new drugs to treat patients with ESCC [1, 2, 3, 6]. During the past few years, metformin has attracted more concern due to its anti-cancer effects [18–21]. However there are fewer report of metformin in ESCC. Therefore understanding its function and the molecular mechanisms which contribute to its function were vital for the treatment of ESCC in future.

Metformin, due to its definite effect, low cost and less adverse reactions has become a first-line medication for treatment of type 2 diabetes mellitus [22, 23]. Evans et al in 2005 firstly reported that metformin could reduce the incidence of cancer in patients with diabetes mellitus [24]. In addition, there were increasing evidences that metabolic reprogramming in malignancy increased the proliferation ability of tumors. So, agents targeting metabolic abnormalities attracted more attention, metformin was at the forefront. A number of meta analyses had found that metformin could reduce the incidence and mortality of cancer in patients with type 2 diabetes, and improve the prognosis of patients with cancer [25, 26]. While it is not clear that whether or not any positive effects could be exert to cancer patients without diabetes mellitus.

Here, we demonstrated that treatment of ESCC cell line KYSE450 with different concentration of metformin could diminish cells growth and it was in a time and dose dependent manner. Dysregulated apoptosis was shown to be involved in the onset and progression of several human cancers. Furthermore, different concentration of metformin could also promote the apoptosis of KYSE450, and it was also in a time and dose dependent manner. Sesen et al reported that metformin significantly reduced human glioma cell proliferation, the number of GB cells undergoing division and enhanced cell cycle arrest [27]. Micic D et al demonstrated that metformin could decrease the incidence and mortality for some certain cancers [28]. Consistent with these previous reports, we showed that metformin inhibited ESCC cell proliferation and increased cell apoptosis efficiently. Metformin anti-cancer effect in vitro led us to test its efficiency in vivo in xenograft tumor of KYSE540 in nude mice. We showed that daily treatment of xenograft tumor of KYSE540 with metformin (5.75 mg/kg/d) markedly reduced tumor growth compared to those treated with normal saline. Most important of all, the mice tolerated with the dosage of metformin we used well and no toxicity was induced during the experiment. So metformin could be a more and more attractive drug for treatment of ESCC in monotherapy.

However, the molecular mechanisms underlying the anti-cancer effect of metformin has not yet been clearly elucidated, particularly in ESCC. A numerous mechanisms had been reported in metformin's anti-cancer effect. mTOR signaling pathway has been suggested to play an critical role in cancer pathogenesis [8,9]. Cancer cell proliferation, survival, and growth required the activation of mTOR signaling. mTORC1 which controlled the protein synthesis process could directly phosphorylates S6K1 and 4EBP1 which promote protein synthesis in turn [8,9]. In this study, we found that after treated with different concentration of metformin (5, 10 and 20 mmol/L), the mRNA and protein expression of S6K1 and 4EBP1 in KYSE450 cells both decreased significantly compared to those in control group. I++t was dose dependent. KYSE450 cells treated with 20 mmol/L metformin for 24, 48, 72h also showed markedly decrease of mRNA and protein expression of S6K1 and 4EBP1, it was time dependent. These results were further confirmed by immunohistochemical analysis of average density on tissues excised from xenograft tumor of KYSE450 cells. The average density of stain both S6K1 and 4EBP1 in xenograft tumor treated with metformin was weaker than those in xenograft tumor treated with normal saline. All these verified that metformin inhibited two key factors of the mTOR signaling pathway, S6K1 and 4EBP1 in vitro and in vivo. Metformin perhaps exerted its anti-cancer effect via reducing S6K1 and 4EBP1 mRNA and protein expression in ESCC.

Chemotherapy remains to be one of the most effective methods for patients with ESCC in middle and late stage [29]. Though chemotherapy had yielded some success, there existed great limitation with regard to side effects, duration of therapy and response rates [6, 7]. So there is increasing interest in using such strategies as combination of drugs. Cisplatin attracted our attention because of its anticancer effects in a variety of cancers. Cisplatin was one of the most compelling chemotherapy drugs among those which were widely used now [29]. While it also had those problems we listed, so cisplatin was often commonly used in combination with others to overcome these problems. In the current study, we demonstrated that combination of metformin with cisplatin had greater anti-cancer effects than those treated with metformin or cisplatin alone. And combination of metformin with cisplatin exerted their effects via inhibiting the proliferation and promoting the apoptosis ability of ESCC cells. As far as we know, this is the first study to demonstrate the combination effects of metformin and cisplatin in ESCC cells.

In summary, this study highlighted a new effect of metformin in its anti-cancer action in ESCC and indicated that metformin inhibited the proliferation and promoted the apoptosis markedly in ESCC cell line: KYSE450. In vivo study also showed that metformin decreased xenograft tumor growth significantly. Additionally, metformin enhanced the anti-cancer effects of cisplatin. For mechanism study, our results demonstrated that metformin could exert its effect via reducing S6K1 and 4EBP1 mRNA and protein expression in vitro and in vivo. These results could provide a solid foundation for the use of metformin to treat patients with ESCC in their middle or late stage in monotherapy or combination therapy.

## Supporting information

S1 Data This is the data of the Fig. (RAR)
